# Attitudes Toward E-Learning Among Undergraduate Nursing Students in Greece: A Multicenter Cross-Sectional Study

**DOI:** 10.7759/cureus.118574

**Published:** 2026-10-25

**Authors:** Panagiotis K Anagnostou, Stefanos Mantzoukas, Konstantinos Poulas, Anastasios Tzenalis, Vasiliki Georgiopoulou, Georgios Vlachopoulos, Nikolaos Bakalis

**Affiliations:** 1 Department of Nursing, School of Health Rehabilitation Sciences, University of Patras, Patras, GRC; 2 Research Laboratory Psychology of Patients, Families and Health Professionals, University of Ioannina, Ioannina, GRC; 3 Laboratory of Molecular Biology and Immunology, University of Patras, Patras, GRC; 4 Department of Medical Physics, School of Medicine, University of Patras, Patras, GRC

**Keywords:** blended learning, digital competence, e-learning, nursing education, nursing students, technology acceptance

## Abstract

Background/objectives

E-learning has become an integral component of higher education, including nursing programs that combine theoretical instruction with laboratory and clinical training. This multicenter study investigated undergraduate nursing students' attitudes toward e-learning in Greece and examined the individual, educational, and technology-related factors associated with these attitudes.

Methods

A multicenter cross-sectional study was conducted among 1,148 undergraduate nursing students from all eight public university nursing departments operating in Greece during the study period. Data were collected using the Greek version of the Attitude toward e-Learning Scale among Nursing Students (ALSNS). Statistical analyses included descriptive statistics, comparative tests, and Bonferroni-adjusted post hoc analyses.

Results

Students generally recognized the flexibility and accessibility of e-learning and expressed positive intentions toward using digital educational technologies. However, they remained cautious regarding fully online approaches as a replacement for conventional nursing education and showed a preference for blended learning approaches. More positive attitudes were associated with younger age, university affiliation, higher digital competence, higher family income, and advanced year of study (p < 0.05), whereas gender differences were negligible (Cohen's d < 0.15).

Conclusions

Undergraduate nursing students viewed e-learning as a valuable complement rather than a replacement for conventional teaching. The findings suggest that blended learning models may be appropriate, integrating digital instruction with face-to-face nursing education while considering differences in students' digital readiness and educational context. Future longitudinal studies should examine whether positive attitudes toward e-learning translate into improved academic outcomes and clinical competence.

## Introduction

Distance education has become one of the most important developments in higher education, particularly following the COVID-19 pandemic, which accelerated the adoption of digital learning worldwide [1]. During periods of educational disruption, universities rapidly implemented synchronous and asynchronous online teaching [2] to ensure academic continuity across healthcare disciplines, including nursing education [3]. Beyond the emergency transition to remote teaching, digital technologies have continued to influence the design and delivery of higher education, raising important questions regarding their long-term integration into nursing curricula.

The integration of information and communication technologies (ICTs) has transformed higher education by expanding access to learning resources, increasing flexibility, and promoting more student-centered educational approaches [4]. Learning management systems (LMS), videoconferencing platforms, and other digital technologies enable continuous access to educational content, facilitate communication between students and faculty [5], and support self-directed learning [6].

Despite these advantages, the implementation of e-learning in nursing education presents unique challenges. Nursing curricula rely heavily on laboratory instruction, clinical placements, and experiential learning, all of which require direct interaction with patients, educators, and healthcare environments [7]. Consequently, concerns remain regarding the ability of fully online learning to support the development of psychomotor skills, clinical reasoning, professional communication, and clinical competence [8]. Although e-learning effectively supports theoretical instruction, it cannot fully replicate the clinical and laboratory components that are fundamental to nursing education [9].

Previous studies consistently report that nursing students value the flexibility, accessibility, and convenience of e-learning, particularly during crisis situations [10]. However, students have also identified important limitations, including reduced interaction with instructors, technological barriers, social isolation, lower engagement, and concerns regarding clinical preparedness [11]. In addition, some studies have reported that prolonged exposure to pandemic-related online learning was associated with increased stress, fatigue, and psychological burden among healthcare students [12].

Current evidence increasingly supports blended learning as the most appropriate educational approach for nursing programs. By combining online instruction with face-to-face teaching, blended learning offers greater flexibility while preserving the clinical, practical, and interpersonal experiences essential for nursing education [13,14]. Students' acceptance of e-learning is shaped by several individual and institutional factors, including age, digital literacy, previous experience with online learning, technological infrastructure, and organizational readiness [15]. These observations are consistent with the Technology Acceptance Model (TAM), which proposes that perceived usefulness and perceived ease of use are key determinants of technology adoption [16]. However, the relative contribution of these factors may vary across educational contexts, making country-specific and multicenter investigations necessary.

Reliable assessment of students' attitudes toward e-learning is essential for identifying factors that facilitate or hinder the successful implementation of digital education. Guillasper et al. [17] evaluated the psychometric properties of an attitude toward e-learning instrument among nursing students. The original attitude items were published by Zabadi and Al-Alawi [18]. In the present study, the Greek adaptation is referred to as the Attitude toward e-Learning Scale among Nursing Students (ALSNS).

Although instruments such as the ALSNS support the systematic assessment of nursing students' attitudes toward e-learning [17,18], evidence from Greece remains limited. Existing studies have primarily been conducted within single institutions or specific student populations, restricting the generalizability of their findings. To the best of our knowledge, no multicenter study has investigated undergraduate nursing students' attitudes toward e-learning across Greek universities or examined their associations with demographic, educational, socioeconomic, and technology-related characteristics in the post-pandemic educational context. As digital technologies become increasingly embedded in undergraduate nursing education, multicenter evidence is needed to better understand the factors influencing students' acceptance of e-learning and to inform the development of effective and equitable blended learning strategies [19].

The primary objective of this study was to assess undergraduate nursing students’ self-reported attitudes toward e-learning across public universities in Greece using the Greek adaptation of the Attitude toward e-Learning Scale among Nursing Students (ALSNS). The secondary objectives were to: (1) describe students’ responses to the ALSNS items addressing perceived usefulness and effectiveness of e-learning, intention to use e-learning technologies, and preferences for online versus conventional teaching; and (2) examine associations between ALSNS item responses and demographic, academic, socioeconomic, and technology-related characteristics, including age, gender, university affiliation, year of study, employment status, family income, marital status, computer knowledge, and the primary device used for e-learning. These analyses were intended to identify patterns of association and were not designed to establish causal relationships.

## Materials and methods

Study design

This multicenter cross-sectional study represented the quantitative component of a broader research project investigating undergraduate nursing students' attitudes toward e-learning in Greece.

Participants and data collection

The study population comprised undergraduate nursing students enrolled in public universities across Greece. Participants were recruited from all eight public university nursing departments operating during the study period: the University of Patras, the University of Thessaly, the University of Ioannina, the National and Kapodistrian University of Athens, the International Hellenic University, the Hellenic Mediterranean University, the University of West Attica, and the University of Peloponnese. These departments represent the full institutional coverage of public university nursing education in Greece. Within each participating institution, students were recruited using a convenience sampling strategy.

Eligible participants were undergraduate nursing students who were actively enrolled during the study period and provided informed consent. Students from all academic years were eligible to participate. As the survey link was disseminated through multiple channels and the total number of eligible students who received the invitation could not be established, the response rate could not be calculated. Questionnaires with incomplete responses were excluded from the final analysis. The final analytical sample comprised 1,148 participants.

Data collection was conducted between July 2021 and February 2022 using a self-administered online questionnaire implemented through the Google Forms platform. The English-language rendering is provided in Appendix 1, which contains the demographic, academic, and background questions; Appendix 2 contains the 11 attitude items. The survey link was distributed through institutional e-mail lists, official student groups, and university learning management systems. Faculty members also informed students about the study during scheduled classes. To reduce the likelihood of duplicate responses, Google Forms was configured to accept a single submission per participant, and additional data-screening procedures were performed to identify duplicate response patterns. Participation was voluntary and anonymous. Electronic informed consent was obtained from all participants before questionnaire completion.

Prior to the main study, a pilot study was conducted with 70 students to assess the clarity and comprehensibility of the questionnaire. Based on the pilot testing, minor linguistic modifications were introduced to improve item clarity and comprehensibility before the main data collection.

Sample size determination

The required sample size was determined a priori using Cochran's formula for cross-sectional studies estimating a population proportion [20]:

$n_0$ = \frac{Z^2 \times p \times (1-p)}{d^2}\)

where:

$n_0$ = initial sample size

*Z*= the z-statistic corresponding to the desired confidence level (1.96 for 95% confidence)

*p*= the estimated proportion of the population (set at 0.50 to maximize the required sample size when the true proportion is unknown)

*d*= the margin of error (set at 0.05, corresponding to ±5%)

Applying these parameters, the initial sample size was estimated. Since the target population of undergraduate nursing students in Greece was finite, the initial sample size was adjusted using the finite population correction formula:

$n_0$=(1.96)^2^ X 0.50 X (1-0.50) / (0.05)^2^ = 3.8416 X 0.25 /0.0025 = 384.16

$n = \frac{n_0}{1+\frac{n_0-1}{N}}$

where N represents the total number of undergraduate nursing students enrolled in Greek public universities, estimated at approximately 8,000-10,000 based on national higher education enrollment data reported by the Hellenic Statistical Authority (ELSTAT) [21]; this yielded an adjusted sample size of approximately 367-370 participants.

To further assess whether the sample would provide adequate statistical power for the planned inferential analyses, an additional power analysis was conducted using GPower software (Heinrich-Heine-Universität Düsseldorf, Düsseldorf, Germany) [22]. Assuming a small effect size ( = 0.10), a significance level of  = 0.05, and statistical power of 0.80, the analysis indicated that a minimum of 600 participants would be required. The final sample of 1,148 participants substantially exceeded both the adjusted sample size estimated using Cochran's formula and the minimum sample size indicated by the GPower analysis, and was therefore considered adequate for the planned analyses.

Pilot testing and item‑level analysis

Prior to the main study, a pilot study was conducted with 70 undergraduate nursing students to assess the clarity, comprehensibility, and preliminary psychometric properties of the Greek version of the ALSNS. Item-level analysis included examination of item means and standard deviations, corrected item-total correlations, and Cronbach’s alpha if an item was deleted. Internal consistency in the pilot sample was satisfactory (Cronbach’s α = 0.827). Exploratory factor analysis was also conducted to examine the preliminary factor structure, yielding a two-factor solution with a reported explained variance of 58.2% and factor loadings ranging from 0.571 to 0.793.

Following review by a three-member expert panel and cognitive debriefing with undergraduate nursing students, two items were reworded to improve clarity and comprehensibility. No items were removed, and all 11 attitude items were retained in the final questionnaire. The revised questionnaire was subsequently administered to the main study sample. The pilot findings were used to inform linguistic refinement; the factor structure and psychometric properties were examined again in the full sample.

Instrument

The instrument consisted of the 11 attitude items originally published by Zabadi and Al-Alawi [18], referred to in this study as the Attitude toward e-Learning Scale among Nursing Students (ALSNS). Guillasper et al. [17] subsequently investigated the psychometric properties of the instrument among nursing students.

The instrument was translated into Greek using a forward-backward translation procedure. Two professional bilingual translators whose native language was Greek independently translated the original English items into Greek. Their translations were compared item by item, and discrepancies were discussed and reconciled. A third independent bilingual translator, who had not participated in the forward translation and had no access to the original items, translated the reconciled Greek version back into English. The research team compared the back-translated version with the original instrument, and discrepancies were resolved by consensus among the translators and the research team.

A three-member expert panel comprising academic nursing faculty reviewed the Greek version for semantic, idiomatic, experiential, and conceptual equivalence. Cognitive debriefing was subsequently conducted with undergraduate nursing students, who were asked to paraphrase the items and identify unclear or ambiguous wording. Based on the expert review and student feedback, two items were reworded to improve clarity and comprehensibility. No items were removed, and the final questionnaire retained all 11 attitude items.

The ALSNS items address perceptions of e-learning, including its perceived usefulness and effectiveness, intention to use e-learning technologies, and preferences regarding online and conventional teaching [17,18]. Responses are recorded on a five-point Likert scale ranging from 1 (“strongly disagree”) to 5 (“strongly agree”). The handling of the negatively worded item is described in the statistical analysis section.

Written permission to use the instrument was obtained from the relevant authors [17,18]. The original publication by Zabadi and Al-Alawi [18] also identifies a Creative Commons Attribution 3.0 licence. The adapted questionnaire items are attributed to the original source, and all data and statistical results reported in this study were generated from the present sample.

Ethical considerations

The study was conducted in accordance with the Declaration of Helsinki for research involving human participants [23] and complied with the European Union General Data Protection Regulation (GDPR) concerning the protection of personal data [24].

Ethical approval was obtained from the Research Ethics and Deontology Committee of the University of Patras (Protocol No. 7601/06-07-2021).

Participation was voluntary and anonymous. Before completing the questionnaire, participants received information regarding the study objectives, confidentiality of responses, voluntary participation, their right to withdraw at any time without consequences, and the intended use of the collected data. Electronic informed consent was obtained from all participants before data collection [25]. Access to the study database was restricted to the research team, and no personally identifiable information was collected.

Statistical analysis

Statistical analyses were performed using IBM SPSS Statistics version 28.0 (IBM Corp., Armonk, NY). Descriptive statistics are presented as frequencies, percentages, means, and standard deviations. Item-level analyses were conducted to examine specific dimensions of students' attitudes toward e-learning as reflected by individual ALSNS items. Independent-samples t-tests were used to compare two-group variables (e.g., gender and employment status), whereas one-way analysis of variance (ANOVA) was performed for variables with more than two categories, including age group, year of study, university affiliation, computer knowledge, family income, place of residence, marital status, number of children, and primary device used for e-learning. When statistically significant differences were identified, Bonferroni-adjusted post hoc comparisons were conducted to control for Type I error arising from multiple pairwise comparisons. Effect sizes were reported using Cohen's d for t-tests and omega squared (ω²) for ANOVA. Statistical significance was defined as p < 0.05.

## Results

Participant characteristics

A total of 1,148 undergraduate nursing students from eight Greek universities participated in the study (Table 1). The sample was predominantly female (82.8%) and comprised mainly students aged 18-23 years (83.0%), reflecting the typical demographic profile of undergraduate nursing students in Greece. Most participants were enrolled in the first three years of their studies, with second-year students representing the largest subgroup (29.5%).

Most respondents resided in urban areas (71.0%), were unmarried (93.7%), and had no children (91.1%). Although the majority were not employed (69.0%), nearly one-third combined university studies with paid employment.

With regard to socioeconomic and technology-related characteristics, almost half of the participants reported an annual family income below €10,000. Digital readiness was high across the sample, with 96.8% reporting reliable internet access and laptops being the most commonly used device for e-learning (65.4%).

The inclusion of students from universities located in different geographical regions increases the diversity of the sample and supports the transferability of the findings to the Greek undergraduate nursing student population.

Table 1 presents the demographic characteristics of the study sample (N = 1,148).

**Table 1:** Demographic characteristics of the study sample (N = 1,148) Note. n = number of participants; % = percentage.

Variable	Category	n	%
Gender	Female	950	82.8
	Male	198	17.2
Age group	18-23 years	953	83
	24-33 years	109	9.5
	34-43 years	36	3.1
	>44 years	50	4.4
University	University of Patras	320	27.9
	University of Thessaly	233	20.3
	University of Ioannina	199	17.3
	National and Kapodistrian University of Athens	121	10.5
	Other universities	275	24
Year of study	1st year	254	22.1
	2nd year	339	29.5
	3rd year	265	23.1
	4th year	135	11.8
	Degree pending	126	11
	Equivalency	29	2.5
Employment status	Not working	792	69
	Working	356	31
Place of Residence	Urban area	815	71
	Rural area	215	18.7
	Island area	118	10.3
Marital status	Unmarried	1076	93.7
	Married	72	6.3
Children	No children	1046	91.1
	One or more children	102	8.9
Annual family income	< €5,000	231	20.1
	€5,001–€10,000	298	26
	€10,001–€20,000	264	23
	Other income categories	355	30.9
Main device used for E-learning	Laptop	751	65.4
	Smartphone	231	20
	Tablet	83	7.3
	Desktop computer	83	7.3

Psychometric properties of the Greek ALSNS

In the pilot sample, the instrument demonstrated satisfactory internal consistency (Cronbach's α = 0.827). The KMO measure was 0.854, and Bartlett's test of sphericity was statistically significant (p < 0.001). Exploratory factor analysis (EFA) using principal axis factoring yielded a two‑factor structure accounting for 58.2% of the initial variance, with factor loadings ranging from 0.571 to 0.793.

In the full sample, the psychometric properties were confirmed and, in most respects, improved. The KMO measure increased to 0.915, indicating excellent sampling adequacy for factor analysis, and Bartlett's test of sphericity remained statistically significant (χ²(55) = 6291.23, p < 0.001). The same two‑factor structure was retained, accounting for 58.90% of the initial variance and 50.28% of the variance after extraction. The first factor explained 45.55% of the variance after extraction, and the second factor explained an additional 4.73%. Internal consistency in the full sample remained satisfactory (Cronbach's α = 0.792), although it was slightly lower than in the pilot sample, a finding consistent with the greater heterogeneity of the larger sample.

Examination of the factor matrix showed that eight of the eleven items loaded strongly on the primary factor, with loadings ranging from 0.587 to 0.869. The reverse‑coded item 10 ("E‑learning is not an effective learning method") loaded negatively on the primary factor (−0.594), as expected given its reverse wording. Two items showed weak loadings in the full sample: item 1 ("I am interested in attending courses that use e‑learning"; loading = 0.228; communality = 0.053) and item 6 ("The e‑learning environment requires advanced technical knowledge for effective use"; loading = −0.012; communality = 0.007). Both items were retained in the scale because they are conceptually relevant to the content domain of the instrument and because the overall scale reliability and factor structure were satisfactory. Their limited contribution to the factor solution is acknowledged as a limitation and discussed below.

Overall, the full‑sample analysis confirmed the two‑factor structure identified in the pilot study, supported the construct validity of the Greek ALSNS, and demonstrated improved sampling adequacy in the larger sample.

The psychometric properties of the Greek version of the ALSNS were evaluated in the pilot sample (n = 70) and re‑examined in the full sample (N = 1,148). The results are summarised in Table 2.

**Table 2:** Psychometric properties of the Greek version of the Attitude Toward e-Learning Scale Among Nursing Students (ALSNS).

Psychometric property	Indicator/method	Pilot Sample (N = 70); value/result	Full sample (N = 1,148); value/result
Internal consistency (reliability)	Cronbach's alpha (α)	0.827	0.792
Sampling adequacy	Kaiser-Meyer-Olkin (KMO)	0.854	0.915
Test of factorability	Bartlett's Test of Sphericity	p < 0.001	χ²(55) = 6291.23, p < 0.001
Construct validity	Exploratory factor analysis (EFA)	Two‑factor structure	Two‑factor structure
Explained variance	Total variance explained	58.20%	58.90%

Descriptive analysis of attitudes toward e-learning

Descriptive statistics for the 11 ALSNS items are presented in Table 3. Item-level mean scores ranged from 2.26 to 3.67, indicating variation in students’ attitudes toward e-learning. The highest mean score was observed for intention to use e-learning tools if available during the semester (Item 4; M = 3.67, SD = 1.04). The lowest mean scores were observed for preference for online courses over face-to-face instruction (Item 7; M = 2.26, SD = 1.23) and overall preference for e-learning over traditional teaching (Item 11; M = 2.30, SD = 1.13). The remaining items showed a range of intermediate mean scores. Overall, the findings suggest that students expressed willingness to use e-learning technologies but were less inclined to prefer online instruction over conventional teaching.

**Table 3:** Descriptive statistics of the ALSNS (N = 1,148). Note. R = indicates a reverse-coded item; Std. Deviation = Standard Deviation; CI = Confidence Interval. The attitude-item wording is adapted from Zabadi and Al-Alawi [18] under the Creative Commons Attribution 3.0 licence (https://creativecommons.org/licenses/by/3.0/). The numerical results are from the present study.

Item	Mean	Std. Deviation	95% CI
Effectiveness of e-learning			
1. I am interested in attending courses that use e-learning	3.24	1.15	(3.17, 3.31)
2. I think that e-learning enhances my learning experiences	2.88	1.15	(2.81, 2.94)
3. Online presentation of courses makes learning more effective	2.66	1.14	(2.59, 2.72)
4. I intend to use e-learning tools if available during the semester	3.67	1.04	(3.61, 3.71)
5. I have a positive opinion about e-learning	3.23	1.14	(3.17, 3.30)
6. The e-learning environment requires advanced technical knowledge for effective use	3.30	1.15	(3.24, 3.37)
7. I would prefer online courses over face-to-face instruction	2.26	1.23	(2.19, 2.34)
8. Online learning is more comfortable and enjoyable	2.88	1.19	(2.82, 2.95)
Preference for e-learning			
9. E-learning is a good alternative to the traditional paper-based system	3.04	1.17	(2.97, 3.11) *
10. E-learning is not an effective learning method (R)	2.79	1.15	(2.72, 2.86)
11. Overall, I prefer e-learning and believe it is better than traditional teaching	2.30	1.13	(2.23, 2.36)

Inferential analysis of factors influencing attitudes toward e-learning

Effect of Age Group

Students' attitudes toward e-learning varied significantly across age groups (Table 4). Significant between-group differences were identified for 10 of the 11 ALSNS items, whereas perceptions regarding the technical knowledge required for using e-learning did not differ significantly among age groups (p = 0.141). The most pronounced differences were observed for preference for online over face-to-face instruction (F(3, 1144) = 19.89, p < 0.001, ω² = 0.047), perceived enhancement of learning experiences (F(3, 1144) = 17.34, p < 0.001, ω² = 0.041), and positive opinion about e-learning (F(3, 1144) = 16.02, p < 0.001, ω² = 0.038). Bonferroni-adjusted post-hoc comparisons showed that students aged 18-23 years expressed more favorable attitudes than those aged 34-43 years and >44 years across several ALSNS items (p < 0.001). Furthermore, the youngest age group demonstrated a stronger preference for online learning than all other age groups (p < 0.01).

**Table 4:** Effect of age group on attitudes toward e-learning (ANOVA results) Note. F = F-statistic; p = significance level; ω² = Omega squared

Item	F(3, 1144)	p-value	ω²
Interest in attending courses using e-learning	2.90	0.034	0.005
E-learning enhances learning experiences	17.34	<0.001	0.041
Online presentation makes learning more effective	14.41	<0.001	0.034
Intention to use e-learning tools if available	5.50	<0.001	0.012
Positive opinion about e-learning	16.02	<0.001	0.038
E-learning requires advanced technical knowledge	1.82	0.141	0.002
Preference for online over face-to-face instruction	19.89	<0.001	0.047
Online learning is more comfortable and enjoyable	9.80	<0.001	0.022
E-learning is a good alternative to traditional system	4.64	0.003	0.009
E-learning is not an effective learning method	6.66	<0.001	0.015
Overall preference for e-learning over traditional teaching	10.31	<0.001	0.024

Effect of University Affiliation

Students' attitudes toward e-learning differed significantly according to university affiliation (Table 5). Significant between-university differences were identified across all ALSNS items. The most pronounced differences were observed for positive opinion about e-learning (F(7, 1140) = 13.75, p < 0.001, ω² = 0.072), perceived enhancement of learning experiences (F(7, 1140) = 13.08, p < 0.001, ω² = 0.069), and perceived effectiveness of online course delivery (F(7, 1140) = 12.15, p < 0.001, ω² = 0.064). Bonferroni-adjusted post-hoc comparisons revealed that students from the University of Patras and the University of Thessaly reported more favorable attitudes toward e-learning than students from several of the other participating universities (p < 0.05).

**Table 5:** Effect of university affiliation on attitudes toward e-learning (ANOVA results). Note. F = F-statistic; p = significance level

Item	F(7, 1140)	p-value	Omega-squared
Interest in attending courses using e-learning	7.82	<0.001	0.040
E-learning enhances learning experiences	13.08	<0.001	0.069
Online presentation makes learning more effective	12.15	<0.001	0.064
Intention to use e-learning tools if available	3.95	<0.001	0.018
Positive opinion about e-learning	13.75	<0.001	0.072
E-learning requires advanced technical knowledge	2.92	0.005	0.012
Preference for online over face-to-face instruction	11.59	<0.001	0.061
Online learning is more comfortable and enjoyable	10.78	<0.001	0.056
E-learning is a good alternative to traditional system	4.52	<0.001	0.021
E-learning is not an effective learning method	8.07	<0.001	0.041
Overall preference for e-learning over traditional teaching	9.62	<0.001	0.050

Effect of Computer Knowledge Level

Students with different levels of computer knowledge exhibited significantly different attitudes toward e-learning (Table 6). Significant between-group differences were identified across all levels of self-reported computer knowledge. The most pronounced differences were observed for positive opinion about e-learning (F(4, 1143) = 13.38, p < 0.001, ω² = 0.045), online learning being comfortable and enjoyable (F(4, 1143) = 11.80, p < 0.001, ω² = 0.040), and intention to use e-learning tools if available (F(4, 1143) = 10.85, p < 0.001, ω² = 0.037). Bonferroni-adjusted post-hoc comparisons indicated that students reporting excellent or very good computer knowledge expressed more favorable attitudes toward e-learning than those reporting good or moderate computer knowledge (p < 0.05). In addition, students with inadequate computer knowledge differed significantly from some intermediate knowledge groups on selected ALSNS items.

**Table 6:** Effect of computer knowledge level on attitudes toward e-learning (ANOVA results) Note. F = F-statistic; p = significance level

Item	F(4, 1143)	p-value	Omega-squared
Interest in attending courses using e-learning	2.805	0.025	0.010
E-learning enhances learning experiences	5.004	<0.001	0.012
Online presentation makes learning more effective	7.655	<0.001	0.026
Intention to use e-learning tools if available	10.852	<0.001	0.037
Positive opinion about e-learning	13.381	<0.001	0.045
E-learning requires advanced technical knowledge	2.791	0.025	0.010
Preference for online over face-to-face instruction	6.986	<0.001	0.024
Online learning is more comfortable and enjoyable	11.804	<0.001	0.040
E-learning is a good alternative to traditional system	7.573	<0.001	0.026
E-learning is not an effective learning method	4.899	<0.001	0.017
Overall preference for e-learning over traditional teaching	9.058	<0.001	0.031

Effect of Year of Study

Year of study was significantly associated with students' attitudes toward e-learning (Table 7). One-way ANOVA revealed significant differences among academic years for nine of the 11 ALSNS items. No significant differences were observed for the items assessing whether e-learning requires advanced technical knowledge (p = 0.075) and whether e-learning is a good alternative to the traditional paper-based system (p = 0.698). The largest effect sizes were observed for positive opinion about e-learning (F(5, 1142) = 9.05, p < 0.001, ω² = 0.038), preference for online over face-to-face instruction (F(5, 1142) = 7.09, p < 0.001, ω² = 0.030), and online learning being comfortable and enjoyable (F(5, 1142) = 6.47, p < 0.001, ω² = 0.028). Bonferroni post-hoc analyses indicated that third- and fourth-year students, as well as students awaiting graduation, reported significantly more positive attitudes toward e-learning compared with first-year students (p < 0.05).

**Table 7:** Effect of year of study on attitudes toward e-learning (ANOVA results) Note. F = F-statistic; p = significance level

Item	F(5, 1142)	p-value	Omega-squared
Interest in attending courses using e-learning	6.14	<0.001	0.026
E-learning enhances learning experiences	3.78	0.002	0.016
Online presentation makes learning more effective	2.68	0.021	0.012
Intention to use e-learning tools if available	3.99	0.001	0.017
Positive opinion about e-learning	9.05	<0.001	0.038
E-learning requires advanced technical knowledge	2.01	0.075	0.009
Preference for online over face-to-face instruction	7.09	<0.001	0.030
Online learning is more comfortable and enjoyable	6.47	<0.001	0.028
E-learning is a good alternative to traditional system	0.60	0.698	0.003
E-learning is not an effective learning method	3.42	0.005	0.015
Overall preference for e-learning over traditional teaching	4.72	<0.001	0.020

Effect of Gender

Independent‑samples t‑tests were conducted to compare male and female students across all 11 ALSNS items. Female students reported slightly higher mean scores than male students on most items; however, none of the differences reached statistical significance, and all effect sizes were negligible (Cohen's d < 0.15). The largest mean differences were observed for item 5 ("I have a positive opinion about e‑learning"; male M = 3.14, SD = 1.11; female M = 3.25, SD = 1.15; t(1146) = −1.28, p = 0.201, d = 0.09) and item 4 ("I intend to use e‑learning tools if available during the semester"; male M = 3.58, SD = 0.95; female M = 3.69, SD = 1.06; t(1146) = −1.38, p = 0.168, d = 0.11). The smallest difference was observed for item 8 ("Online learning is more comfortable and enjoyable"; male M = 2.90, SD = 1.14; female M = 2.88, SD = 1.21; t(1146) = 0.25, p = 0.802, d = 0.01). Complete results for all items are presented in Table 8. Taken together, these findings indicate that gender was not meaningfully associated with attitudes toward e‑learning in this sample.

**Table 8:** Gender differences in attitudes toward e-learning across the 11 ALSNS items (N = 1,148).

Item	Male mean (SD)	Female mean (SD)	t(1146)	p-value	Cohen's d
Interest in attending courses using e-learning	3.15 (1.11)	3.26 (1.17)	−1.26	0.208	0.08
E-learning enhances learning experiences	2.90 (1.17)	2.75 (1.12)	−1.73	0.083	0.13
Online presentation makes learning more effective	2.63 (1.17)	2.66 (1.13)	−3.25	0.745	0.02
Intention to use e-learning tools if available	3.58 (0.95)	3.69 (1.06)	−1.38	0.168	0.11
Positive opinion about e-learning	3.14 (1.11)	3.25 (1.15)	−1.28	0.201	0.09
E-learning requires advanced technical knowledge	3.25 (1.18)	3.31 (1.15)	−0.65	0.518	0.04
Preference for online over face-to-face instruction	2.20 (1.18)	2.28 (1.24)	−0.76	0.446	0.05
Online learning is more comfortable and enjoyable	2.90 (1.14)	2.88 (1.21)	0.25	0.802	0.01
E-learning is a good alternative to traditional system	3.16 (1.17)	3.02 (1.18)	1.59	0.111	0.13
E-learning is not an effective learning method	2.91 (1.14)	2.76 (1.15)	1.66	0.097	<0.01
Overall preference for e-learning over traditional teaching	2.29 (1.13)	2.30 (1.20)	−0.47	0.096	0.13

Additional factors

Effect of Family Income

Students' attitudes toward e-learning varied significantly across family income categories. One-way ANOVA identified significant between-group differences for most ALSNS items. The most pronounced differences were observed for overall preference for e-learning over traditional teaching (F(6, 1141) = 6.95, p < 0.001, ω² = 0.029), preference for online over face-to-face instruction (F(6, 1141) = 6.06, p < 0.001, ω² = 0.031), and online learning being comfortable and enjoyable (F(6, 1141) = 5.62, p < 0.001, ω² = 0.029). Bonferroni-adjusted post-hoc comparisons showed that students from higher-income households expressed more favorable attitudes toward e-learning than those from lower-income households (p < 0.05).

Primary Device Used for E-learning

Attitudes toward e-learning also differed according to the primary device used for online learning. The greatest between-group difference was found for overall preference for e-learning over traditional teaching (F(3, 1144) = 12.16, p < 0.001, ω² = 0.031). Post-hoc analyses using the Bonferroni correction demonstrated that tablet users reported more favorable attitudes than laptop users (p < 0.001), whereas students primarily using smartphones expressed less favorable attitudes than those using laptops (p < 0.05).

Marital Status

Marital status was also associated with students' attitudes toward e-learning. Independent-samples t-tests demonstrated that unmarried students reported more favorable perceptions than married students on several ALSNS items, including perceived enhancement of learning experiences (t(1146) = −3.47, p < 0.001), positive opinion about e-learning (t(1146) = −3.47, p < 0.001), and preference for online over face-to-face instruction (t(1146) = −3.37, p = 0.002).

## Discussion

The present multicenter study examined undergraduate nursing students' attitudes toward e-learning across Greek universities and demonstrated that acceptance of e-learning is associated with multiple individual characteristics, educational contexts, and technology-related factors. Although students acknowledged the flexibility, accessibility, and convenience of e-learning, they remained reluctant to consider it a complete substitute for conventional face-to-face education. Instead, the findings indicate that e-learning is primarily perceived as a complementary educational approach, particularly for theoretical instruction, while in-person teaching continues to be regarded as essential for developing clinical and practical competencies. These results are consistent with the evolving role of digital education in nursing programs following the COVID-19 pandemic, where e-learning has increasingly been incorporated into curricula without replacing experiential clinical training [26,27].

One of the most notable findings was the coexistence of positive attitudes toward digital educational technologies and reservations regarding their perceived educational effectiveness. Although many students expressed willingness to participate in courses incorporating e-learning and reported positive intentions to use digital learning tools, a substantial proportion selected neutral responses when evaluating the effectiveness of online education. This pattern suggests uncertainty rather than rejection of e-learning. Similar findings have been reported internationally, where nursing students recognize the organizational advantages of online education while expressing concerns regarding interaction with instructors, learner engagement, and the acquisition of clinical competencies [28]. Collectively, these findings support the view that students distinguish between the practical benefits of e-learning and its perceived ability to achieve learning outcomes comparable to those of conventional instruction.

Age was significantly associated with attitudes toward e-learning, with younger students demonstrating more favorable perceptions across several dimensions of the ALSNS. Participants aged 18-23 years consistently reported greater acceptance of e-learning than older students, a finding that is consistent with previous studies linking younger age with higher levels of digital familiarity and technology acceptance [29]. Although the concept of "digital natives" has been debated in educational research, younger students often report greater familiarity with digital technologies due to earlier and more frequent exposure to digital environments [30]. These findings suggest that age-related differences in digital confidence should be considered when implementing e-learning initiatives, particularly among mature students who may benefit from additional technical support and structured orientation.

Institutional differences were also observed as an important factor associated with students' attitudes toward e-learning. Participants from different universities demonstrated significant differences in their perceptions of digital learning, suggesting that institutional characteristics may contribute to variations in students' experiences with e-learning. These differences may reflect variations in technological infrastructure, institutional support, previous exposure to digital learning environments, or educational practices. Effective learning management systems, reliable technological resources, and accessible academic support services may enhance students' confidence in digital learning and improve their overall educational experience [31]. The moderate effect sizes observed for university affiliation further suggest that organizational factors should be considered when interpreting differences in students' acceptance of e-learning across institutions [32].

Self-reported computer knowledge was significantly associated with attitudes toward e-learning. Students reporting higher levels of digital competence demonstrated more favorable perceptions across multiple attitude domains, including perceived usefulness, intention to use e-learning, and overall acceptance of e-learning. These findings are consistent with the Technology Acceptance Model, which proposes that perceived ease of use and self-efficacy are important determinants of technology acceptance [16]. Students with greater digital competence may experience fewer technical barriers, engage more confidently with learning platforms, and adapt more readily to technology-enhanced educational environments [33]. From an educational perspective, these findings reinforce the importance of strengthening students' digital competencies alongside the continued expansion of online and blended learning initiatives.

Socioeconomic characteristics were also associated with students' attitudes toward e-learning. Participants from higher-income households reported more favorable perceptions than those from lower-income families, suggesting that financial resources may contribute to differences in students' digital learning experiences. Although most participants reported reliable internet access, socioeconomic differences may still be related to access to appropriate devices, suitable study environments, or other resources that facilitate effective participation in online education. These factors were not directly assessed in the present study; however, previous research has highlighted the potential role of socioeconomic inequalities in shaping engagement with digital learning environments [34,35]. Addressing such inequalities through institutional policies that promote equitable access to technological resources and academic support may help reduce barriers to participation in digital education.

Academic year was significantly associated with students' attitudes toward e-learning. Senior students generally expressed more positive perceptions of e-learning than first-year students, possibly reflecting greater familiarity with university learning environments, increased exposure to digital educational technologies, and more developed self-regulated learning skills [36,37]. As students progress through their studies, they may become more confident in managing online learning demands and integrating digital resources into their academic routines. These findings highlight the importance of providing structured orientation and ongoing digital support, particularly for students entering undergraduate nursing programs.

Although several demographic and educational characteristics were statistically associated with attitudes toward e-learning, the magnitude of these differences was generally small. This suggests that acceptance of digital learning is a multifactorial phenomenon influenced by a combination of personal experiences, technological confidence, and educational context rather than by any single demographic characteristic.

In contrast, gender was not meaningfully associated with attitudes toward e-learning. Although minor differences in mean scores were observed, the corresponding effect sizes were negligible, indicating limited practical significance. This finding is consistent with an increasing body of evidence suggesting that gender differences in technology acceptance have become less pronounced as digital technologies have become increasingly integrated into higher education [38,39]. Nevertheless, the predominance of female participants, reflecting the demographic composition of undergraduate nursing programs, should be considered when interpreting these findings.

Family-related characteristics were also associated with students' perceptions of e-learning. Married students reported less favorable attitudes than unmarried students across several ALSNS dimensions. These differences may reflect the challenges associated with balancing academic responsibilities with family obligations, including reduced availability of study time, competing responsibilities, and increased demands for time management [40]. Flexible educational strategies, including asynchronous learning activities and adaptable course structures, may provide additional support for students with greater personal responsibilities.

An important observation of the present study was that students distinguished between the convenience of e-learning and its perceived educational effectiveness. While digital learning was widely recognized for its flexibility and accessibility, students were less likely to perceive it as superior to conventional face-to-face education. This distinction is particularly relevant in nursing education, where laboratory training, clinical placements, simulation-based learning, and direct interaction with patients remain essential components of professional preparation [41]. The relatively high proportion of neutral responses across several questionnaire items further suggests that students' attitudes reflect a balanced evaluation of the advantages and limitations of e-learning rather than simple acceptance or rejection, indicating that improvements in instructional design and learner engagement may increase acceptance of digital learning environments [42].

Consistent with previous international research, participants demonstrated a preference for blended learning approaches rather than exclusively online education [43]. Hybrid educational models combine the flexibility of digital learning with the interpersonal interaction, immediate feedback, and experiential learning opportunities that are fundamental to nursing education [44]. Rather than viewing e-learning and face-to-face teaching as competing approaches, these findings support their complementary integration within undergraduate nursing curricula. Blended learning may therefore represent an appropriate educational model for combining theoretical knowledge acquisition with the development of clinical competence and professional skills.

Taken together, the findings support the continued integration of blended learning within undergraduate nursing curricula. Digital learning should be incorporated strategically to complement face-to-face education, particularly for theoretical content, independent learning, and asynchronous educational activities. Clinical education, laboratory training, simulation-based learning, and supervised practical experience remain fundamental components of nursing education and are unlikely to be effectively replaced by fully online approaches [45,46]. Future educational strategies should therefore focus on optimizing the complementary strengths of both instructional modalities while preserving the experiential nature of nursing training.

The findings of the present study also have important implications for nursing education. Although participants generally demonstrated positive attitudes toward digital learning, technological accessibility alone may not be sufficient to ensure favorable perceptions of e-learning. Despite widespread internet access and regular use of digital technologies, many students continued to question the educational effectiveness of fully online instruction. These findings suggest that successful implementation of e-learning depends not only on technological infrastructure but also on pedagogically sound instructional design, meaningful interaction between students and educators, active student engagement, and faculty preparedness [16]. Furthermore, the findings reinforce the need to move beyond the emergency remote teaching practices adopted during the COVID-19 pandemic toward intentionally designed digital education. The rapid transition to online learning during the pandemic was primarily driven by necessity rather than educational planning, and students' perceptions may therefore partly reflect experiences associated with emergency remote teaching rather than with well-designed e-learning environments [47,48]. Consequently, the present findings should be interpreted within the context of the post-pandemic educational transition, where students' attitudes may continue to be influenced by their previous experiences of emergency online education.

Strengths and limitations

This study has several important strengths. It is one of the largest multicenter studies conducted in Greece to examine undergraduate nursing students' attitudes toward e-learning, including 1,148 participants from all eight public university nursing departments operating in Greece during the study period, thereby providing nationwide institutional coverage of undergraduate nursing education. The large sample size increased the precision of estimates and enabled comparisons across multiple demographic and educational subgroups. In addition, the assessment of the Greek version of the Attitudes toward e-Learning Scale among Nursing Students (ALSNS), including evaluation of internal consistency and construct validity indicators, provides evidence of satisfactory psychometric indicators and strengthens the interpretability of the findings. Furthermore, reporting effect sizes alongside statistical significance provides a more comprehensive understanding of the practical relevance of observed differences.

Several limitations should also be acknowledged. First, the cross-sectional design does not allow causal conclusions to be drawn, and the identified relationships should be interpreted as associations rather than evidence of cause-and-effect relationships. Second, participants were recruited using convenience sampling, which may have introduced selection bias and limits the extent to which the findings can be generalized beyond the participating universities. Third, all data were collected through self-reported questionnaires and may therefore be influenced by recall bias, response bias, or social desirability bias.

Although students from multiple universities and geographical regions were included, institutional differences in recruitment procedures and educational contexts may have influenced responses. Furthermore, the predominance of female participants reflects the demographic composition of undergraduate nursing programs but may limit the applicability of the findings to populations with a more balanced gender distribution. Also, data collection occurred shortly after the COVID-19 pandemic; therefore, students' attitudes may partly reflect their experiences with emergency remote teaching rather than their perceptions of systematically designed and pedagogically optimized e-learning environments.

An additional limitation concerns the large number of item-level statistical comparisons performed across demographic, academic, socioeconomic, and technology-related variables. Multiple testing increases the probability of false-positive findings (Type I error), particularly when numerous outcomes are examined. Although Bonferroni-adjusted post-hoc comparisons were used following significant ANOVA results, this procedure does not necessarily control the overall error rate across all separate item-level analyses. Accordingly, the findings should be interpreted cautiously, with particular emphasis on the magnitude of the effect sizes rather than statistical significance alone.

Future research should employ longitudinal and mixed-methods approaches to examine how attitudes toward e-learning develop over time and whether these perceptions are associated with academic outcomes, clinical competence, and professional preparedness. Further investigation should also explore the contribution of instructional design, digital self-efficacy, faculty readiness, and institutional support to students' acceptance and effective use of technology-enhanced learning in nursing education.

## Conclusions

The findings of this multicenter study examining undergraduate nursing students’ self-reported attitudes toward e-learning in Greece indicate that the students expressed willingness to use e-learning technologies but were generally less inclined to prefer online instruction over conventional face-to-face teaching. This pattern suggests that positive attitudes toward the use of digital technologies do not necessarily imply acceptance of fully online education as a replacement for traditional teaching. Attitudes toward e-learning were associated with demographic, academic, socioeconomic, and technology-related characteristics, although the observed differences were generally small.

Future longitudinal and intervention-based studies should investigate whether students’ attitudes are associated with engagement, academic performance, and clinical learning outcomes, and whether appropriately designed blended learning approaches provide measurable educational benefits.

## Appendices

Appendix 1

Table 9 contains the questionnaire, which was administered in Greek and comprised demographic, academic, and background questions. An English-language version is presented below.

**Table 9:** Demographic, academic and background characteristics Credits: Research Team.

Question	Response format/options
Age	Open response (years)
Gender	Male; Female
Semester of study	Open response
University at which you are enrolled	University of Patras; University of Ioannina; International Hellenic University; National and Kapodistrian University of Athens; University of the Peloponnese; Hellenic Mediterranean University; University of West Attica; University of Thessaly
Place of permanent residence	Urban area; Island area; Rural area
Marital status	Married; Single
Number of children	None; One; Two; Three or more
Annual family income	Less than €5,000; €5,001-€10,000; €10,001-€20,000; €20,001-€30,000; €30,001-€40,000; More than €40,001
English-language certification	B1; B2 (Lower); C1 (Advanced); C2 (Proficiency)
Are you currently employed?	Yes; No
Average daily Internet use	Open response (hours per day)
Is an Internet connection available at your place of residence?	Yes; No
Primary device used for e-learning	Desktop computer; Laptop computer; Tablet; Smartphone (mobile phone)
How would you rate your knowledge of computer use?	Insufficient; Moderate; Good; Very good; Excellent

Appendix 2

Table 10 contains the 11-item Attitude toward e-Learning Scale among Nursing Students (ALSNS) (Table 10).

**Table 10:** Attitude Toward e-Learning Scale among Nursing Students (ALSNS). Instructions: Please select one response for each statement: 1 = Strongly disagree, 2 = Disagree, 3 = Neither agree nor disagree, 4 = Agree, and 5 = Strongly agree. Source: Adapted from the 11 attitude items in Zabadi and Al-Alawi [18], Table 2, p. 292 (doi:10.5539/ijbm.v11n6p286). Copyright is retained by the original authors; their publication states a Creative Commons Attribution 3.0 licence (https://creativecommons.org/licenses/by/3.0/). Adaptation involved Greek translation, cultural adaptation, and minor wording changes; this table provides an English-language rendering of the questionnaire used. Guillasper et al. [17] are cited for subsequent psychometric evaluation among nursing students, not as the original developers of the items.

No.	Statement	Response
1	I am interested in taking courses that incorporate e-learning.	1 2 3 4 5
2	I believe that e-learning enhances my learning experiences.	1 2 3 4 5
3	Presenting courses online makes learning more effective.	1 2 3 4 5
4	I intend to use e-learning tools during the semester, if available.	1 2 3 4 5
5	I have a positive view of e-learning.	1 2 3 4 5
6	Effective use of an e-learning environment requires advanced technical knowledge.	1 2 3 4 5
7	I would prefer taking courses online rather than in a classroom or face-to-face setting.	1 2 3 4 5
8	Online learning is more comfortable and enjoyable.	1 2 3 4 5
9	E-learning is a good alternative to a paper-based system.	1 2 3 4 5
10	E-learning is not an effective learning method.	1 2 3 4 5
11	Overall, I prefer e-learning and believe that it is better than traditional teaching.	1 2 3 4 5
